# Risk Factors for Massive Intraoperative Blood Loss During Posterior Spinal Instrumentation and Fusion in Children: A Retrospective Cohort Study

**DOI:** 10.3390/children13050671

**Published:** 2026-05-12

**Authors:** Shanshan Zhang, Zhengzheng Gao, Jing Hu, Yi Ren, Xiaohuan Cui, Lijing Li, Jianmin Zhang, Fang Wang

**Affiliations:** Department of Anesthesiology, Beijing Children’s Hospital, Capital Medical University, National Center for Children’s Health, Beijing 100045, China; 18810671859@163.com (S.Z.); sunbrightangel@sina.com (Z.G.); doctor_hj@icloud.com (J.H.); renyi1119@outlook.com (Y.R.); cui_xiaohuan@yeah.net (X.C.); amber1717@163.com (L.L.); zhangjianmin@bch.com.cn (J.Z.)

**Keywords:** scoliosis, posterior spinal instrumentation and fusion, massive intraoperative blood loss, risk factors, children

## Abstract

**Highlights:**

**What are the main findings?**

• Younger age, history of heart disease, a greater number of fused levels, and longer operative time are independent risk factors for massive intraoperative blood loss (≥30% of estimated blood volume) in children undergoing posterior spinal instrumentation and fusion for scoliosis.
• Massive intraoperative blood loss is associated with longer postoperative hospital stay and a higher rate of postoperative allogeneic blood transfusion.

**What are the implications of the main findings?**

• Early identification of high-risk pediatric patients using these factors can guide perioperative blood conservation strategies and optimize anesthetic management to reduce massive bleeding.
• Children with preexisting heart disease require particularly vigilant preoperative cardiopulmonary evaluation and intraoperative monitoring to mitigate the substantially increased risk of massive hemorrhage.

**Abstract:**

**Background:** To investigate the risk factors for and prognostic implications of massive blood loss during posterior spinal instrumentation and fusion (PSIF) in pediatric patients with scoliosis. **Methods:** We retrospectively analyzed the electronic medical records of 460 children who underwent scheduled PSIF under general anesthesia between June 2021 and January 2024. Patients were grouped based on intraoperative blood loss: massive (estimated blood loss [EBL]/estimated blood volume [EBV] ≥ 30%) and nonmassive (EBL/EBV < 30%). Perioperative parameters were compared. Univariate and multivariate logistic regression analyses were performed to identify independent risk factors for massive intraoperative blood loss. **Results:** Among the 460 patients with scoliosis who underwent PSIF, 188 were male and 272 were female (mean age 9.4 ± 4.1 years). Massive intraoperative blood loss occurred in 126 (27%) patients. Factors associated with massive blood loss included age, preoperative Cobb angle, history of heart disease or neurofibromatosis, number of previous scoliosis surgeries, operative time, number of fused levels, number of pedicle screws inserted, and whether osteotomy was performed. Multivariate analysis identified younger age (odds ratios [OR] = 0.829, 95% confidence interval [CI], 0.751–0.914, *p* < 0.001), history of heart disease (OR = 4.338, 95% CI: 1.637–11.498, *p* = 0.003), greater number of fused levels (OR = 1.118, 95% CI: 1.014–1.233, *p* = 0.025), and longer operative time (OR = 1.008, 95% CI: 1.005–1.012, *p* < 0.001) as independent risk factors. Additionally, the massive blood loss group had a longer postoperative hospital stay (*p* = 0.008) and a higher rate of postoperative allogeneic blood transfusion (7.1% vs. 1.2%, *p* = 0.002) than the nonmassive blood loss group. **Conclusions:** Younger age, preexisting heart disease, a greater number of fused levels, and longer operation duration are independent risk factors for massive intraoperative blood loss in children undergoing PSIF for scoliosis.

## 1. Introduction

Scoliosis is a common clinical issue during childhood development and can have congenital, idiopathic, neuromuscular, or other causes [1,2]. Idiopathic scoliosis is the most prevalent in children (approximately 80% of cases [2]) and often requires surgery. Posterior spinal instrumentation and fusion (PSIF) is the most commonly used surgical technique for treating scoliosis [3,4,5]. Despite advancements in surgical and anesthetic management, the incidence of massive intraoperative blood loss remains high during PSIF because of extensive soft tissue exposure, bone resection, and lower total blood volume in children than in adults [2,6]. Consequently, the need for blood transfusions increases.

However, massive blood loss and transfusion can elevate the risk of perioperative complications (e.g., infection, anemia, hypotension, and impaired organ function [7,8,9]), affecting patient outcomes. Therefore, identifying risk factors associated with massive intraoperative blood loss in pediatric PSIF is crucial for developing optimal blood conservation strategies and perioperative anesthetic management. Early recognition of high-risk patients and implementation of appropriate blood-saving measures can reduce blood loss and prevent perioperative complications.

According to previous studies [10,11,12,13], blood loss during PSIF is associated with multiple factors, including preoperative Cobb angle, osteotomy, and number of fused levels. Recent investigations have reinforced the roles of operative time [14,15,16] as consistent contributor to perioperative bleeding in idiopathic, neuromuscular, and congenital scoliosis. Liu et al. [15] demonstrated that older age was an independent predictor of greater absolute perioperative blood loss in 108 children with congenital scoliosis. However, these studies were predominantly focused on procedure-related variables and had a relatively small sample size, comprehensive risk factor models incorporating both surgical and patient-specific variables (e.g., age and pre-existing comorbidities) remain lacking.

Hence, this large-scale retrospective study aimed to comprehensively evaluate the risk factors for and prognostic impact of massive intraoperative blood loss in pediatric patients undergoing PSIF for scoliosis, with a particular focus on under-investigated patient-specific factors such as age and comorbidities.

## 2. Materials and Methods

### 2.1. Patient Selection

This retrospective cohort study was approved by the Ethics Committee of Beijing Children’s Hospital (Approval No.: [2025]-E-117-R). Clinical data were collected from patients with scoliosis who underwent PSIF under general anesthesia between June 2021 and January 2024. Inclusion criteria were diagnosis of scoliosis, scheduled PSIF under general anesthesia, age ≤ 18 years, and American Society of Anesthesiologists (ASA) physical status I–IV. Exclusion criteria were incomplete clinical data, combination with other surgeries, and interruption of surgery.

Of the 542 patients who underwent PSIF during the study period, 47 (8.7%) were excluded due to incomplete clinical records. The reasons for missing data—including incomplete anesthesia charts and missing laboratory results—were administrative in nature and considered unrelated to patient characteristics or outcomes; a complete-case analysis was therefore performed. To minimize selection bias, all consecutive patients meeting the predefined inclusion and exclusion criteria were enrolled, and all procedures were performed by a stable surgical team using standardized institutional protocols.

Massive intraoperative blood loss was defined as estimated blood loss (EBL)/estimated blood volume (EBV) ≥ 30%, where EBV (mL) = 70 mL × body weight (kg) [6]. Based on this definition, patients were divided into two groups: the massive blood loss group (EBL/EBV ≥ 30%) and the nonmassive blood loss group (EBL/EBV < 30%).

### 2.2. Surgical and Anesthetic Management

Senior orthopedic surgeons performed all surgeries. The procedure included exposure, screw placement, release, correction, decortication, bone grafting, and fusion. Blood conservation measures included discontinuation of antiplatelet and anticoagulant medications 1 week preoperatively, preoperative screening and correction of coagulation disorders, and routine intraoperative use of autologous blood recovery. Anesthesia was induced with propofol (2–2.5 mg/kg), sufentanil (0.5–0.7 μg/kg), and cisatracurium (0.15 mg/kg) or rocuronium (0.5 mg/kg). Maintenance was achieved with propofol (4–8 mg·kg^−1^·h^−1^) and remifentanil (0.25–0.50 μg·kg^−1^·h^−1^). Intraoperative fluid management included crystalloids (e.g., compound sorbitol injection and compound sodium chloride) and colloids (e.g., hydroxyethyl starch). Transfusion triggers were based on EBL/EBV ratio and hemoglobin (Hb) levels: crystalloids alone if EBL/EBV < 10%; colloids and crystalloids if 10% ≤ EBL/EBV ≤ 14% and Hb > 80 g/L; allogeneic red blood cells if EBL/EBV > 14% and Hb < 80 g/L, or Hb < 100 g/L with hemodynamic instability. Fresh frozen plasma was administered when massive transfusion was required, at a plasma-to-red blood cell ratio of ≥ 1:2. Anesthesiologists adjusted management based on dynamic blood gas analysis, blood loss, and vital signs to maintain Hb levels above 80 g/L. Transfusion criteria were Relatively standardized and homogeneously applied across all patients by the dedicated pediatric anesthesia team throughout the study period.

Standard monitoring included heart rate (HR), blood oxygen saturation, invasive arterial pressure, temperature, urine output, blood gas analysis, and neuromonitoring (somatosensory and motor evoked potentials). Acid-base balance and electrolytes were corrected as needed, and vasoactive drugs were used to maintain mean arterial pressure (MAP) and HR within ±20% of baseline. During the critical phases of the procedure (e.g., soft tissue dissection, decortication, and osteotomies), anesthesiologists were allowed to use controlled hypotension to maintain MAP at the lower end of this range (i.e., up to 20% below baseline) for minimizing surgical bleeding. Controlled hypotension was not mandated by a uniform protocol but was applied selectively based on clinical judgment, with patient safety ensured by continuous intraoperative neuromonitoring.

Intraoperative EBL was calculated using a combination of three approaches: (i) the volume of blood collected in the surgical suction canister minus any irrigating fluid used intraoperatively, (ii) the net weight increase in surgical sponges used during the procedure, and (iii) the volume of intraoperative salvaged blood processed by the autologous blood recovery system. The circulating nurse and attending anesthesiologist jointly verified and recorded EBL during and at the end of the surgical procedure before wound closure, reaching a consensus on the final value.

### 2.3. Data Collection

Perioperative data were collected from electronic medical records and anesthesia charts. Preoperative data included age, sex, body mass index (BMI), ASA class, preoperative Hb, hematocrit (Hct), maximum Cobb angle measured from standing full-spine radiographs, history of heart disease or neurofibromatosis, and number of previous scoliosis surgeries. Intraoperative data included surgical details (number of fused levels, whether osteotomy was performed, number of pedicle screws), operative time (skin incision to closure), EBL, volumes of blood products transfused (including autologous blood, allogeneic red cells, and fresh frozen plasma), use of tranexamic acid (TXA; 10 mg/kg loading dose before incision, followed by 5 mg·kg^−1^·h^−1^ maintenance until end of surgery), and urine output. Postoperative data included postoperative destination, length of postoperative hospital stay, need for postoperative transfusion, complications, postoperative Hb and Hct levels, postoperative Cobb angle, and correction rate.

The corrected height was calculated [corrected height = measured height + Y; logY = 0.011X − 0.177, where Y (cm) is height loss due to spinal deformity and X is the maximum preoperative Cobb angle [17]. BMI was calculated as weight (kg) divided by corrected height (m)^2^. Postoperative transfusion was indicated if Hb levels were < 80 g/L or 80–100 g/L, with symptoms of anemia. The correction rate was calculated as (preoperative Cobb angle − postoperative Cobb angle)/preoperative Cobb angle × 100%.

### 2.4. Statistical Analysis

Data were analyzed using SPSS 25.0. Normality was assessed using histograms and the Kolmogorov–Smirnov test. Normally distributed continuous data are presented as mean ± standard deviation and compared using an independent samples t-test. Non-normally distributed data were presented as median (Q1, Q3) and compared using the Mann–Whitney U test. Categorical variables were presented as frequencies and percentages and compared using the χ^2^ test or Fisher’s exact test. Univariate analysis was used to identify potential risk factors for massive blood loss (*p* < 0.1), including age, preoperative Cobb angle, history of heart disease or neurofibromatosis, number of previous scoliosis surgeries, operative time, number of fused levels, number of pedicle screws, and whether osteotomy was performed. These factors were included in a multivariate logistic regression model to identify independent risk factors. Statistical significance was set at *p*-value < 0.05.

## 3. Results

**Figure 1:**
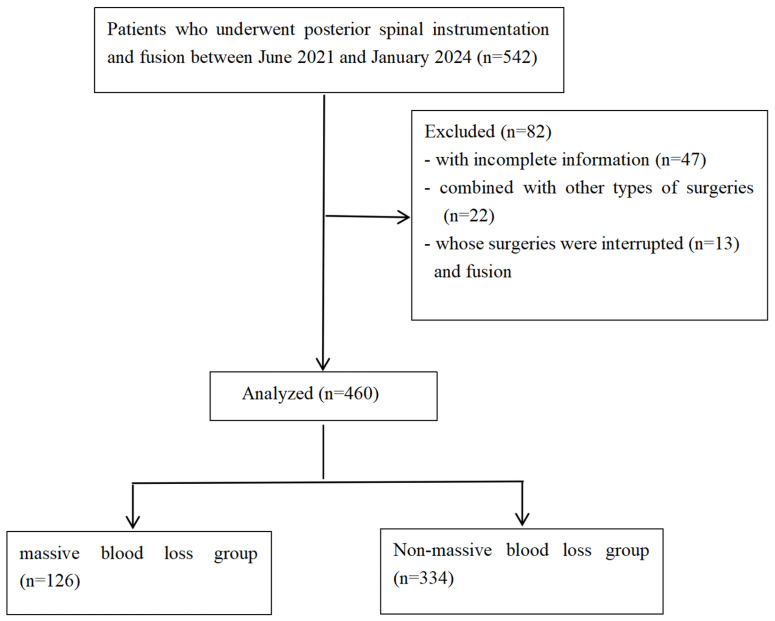
Flow diagram of patient inclusion and allocation.

Between June 2021 and January 2024, 542 patients underwent PSIF. After excluding 47 cases with incomplete data, 22 cases in which other surgeries were also performed, and 13 cases in which surgery was paused owing to decreased spinal cord signals on neurophysiological monitoring or severe hypotension in the prone position, 460 patients with scoliosis were included in the final analysis (Figure 1). Among them, 188 were male and 272 were female (mean age 9.4 ± 4.1 years). Massive intraoperative blood loss occurred in 126 (27.4%) patients. Sex, corrected height, weight, BMI, ASA class, or preoperative Hb or Hct level was not significantly different between groups (Table 1).

Heart disease was present in 11 (3.3%) and 12 (9.5%) patients in the non-massive and massive blood loss groups, respectively (Table 1). In the non-massive blood loss group, the 11 patients with heart disease included atrial septal defect (ASD, *n* = 4), ventricular septal defect (VSD, *n* = 5), patent ductus arteriosus (PDA, *n* = 1), and combined ASD + VSD (*n* = 1). Among them, 1 patient with ASD and 1 with VSD had not undergone surgical repair, while the remaining 9 patients had received corrective cardiac surgery. In the massive blood loss group, the 12 patients included ASD (*n* = 5), VSD (*n* = 1), PDA (*n* = 3), tetralogy of Fallot (TOF, *n* = 1), and combined ASD + VSD (*n* = 2). Among them, 2 patients with ASD and 1 with PDA had not undergone surgical repair, while the remaining 9 patients had received corrective cardiac surgery.

All surgeries were performed by one of three senior pediatric orthopedic spine surgeons (each with > 10 years of experience). There was no statistically significant difference in the rate of massive blood loss among the three surgeons (28.7%, 28.2%, and 25.9%, respectively; χ^2^ = 0.357, *p* = 0.837) (Table 2). And surgeon identity was not retained as a significant variable in the multivariate analysis, indicating that blood loss was primarily driven by patient- and procedure-related factors rather than by individual surgeon differences.

Univariate analysis showed that age, preoperative Cobb angle, history of heart disease or neurofibromatosis, number of previous scoliosis surgeries, number of fused levels, number of pedicle screws, whether osteotomy was performed, and operative time were potential risk factors for massive intraoperative blood loss and were included in the multivariate logistic regression analysis (Table 1 and Table 3). For hemodynamic stability, the massive blood loss group received more allogeneic red blood cells and plasma and had a higher allogeneic transfusion rate intraoperatively than the nonmassive blood loss group (*p* < 0.001) (Table 3). Additionally, the massive blood loss group had a longer postoperative hospital stay (*p* = 0.008) and a higher postoperative transfusion rate (*p* = 0.002). The rate of postoperative allogeneic transfusion was 5.9 percentage points higher in the massive blood loss group. Regarding postoperative complications, no significant differences were observed in postoperative subcutaneous effusion (*p* = 0.706) or pulmonary complications (*p* = 0.369) between groups (Table 4). Two patients (1.6%) in the massive blood loss group were transferred to the intensive care unit (ICU) postoperatively. One patient was directly returned to the ICU with an endotracheal tube because of unstable hemodynamics; the other underwent another endotracheal intubation owing to respiratory failure after endotracheal tube removal and was then transferred to the ICU. All patients in the nonmassive blood loss group returned to the general ward. Analysis of the postoperative destinations of the groups showed no significant differences (*p* = 0.075).

**Figure 2:**
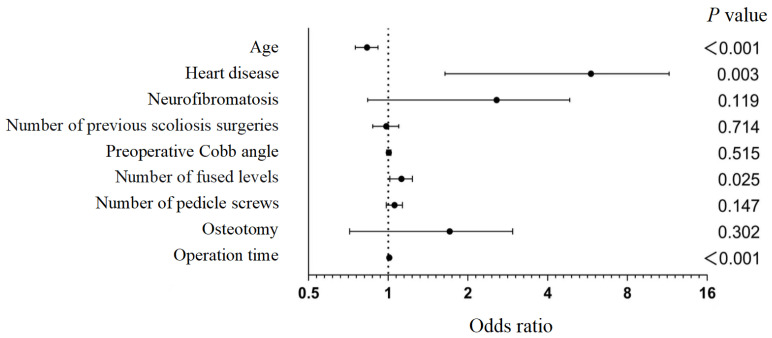
The forest plot shows the independent risk factors that affect the occurrence of massive blood loss during spinal curvature surgery. This forest plot illustrates the influence of various independent risk factors on the occurrence of massive blood loss during spinal curvature surgery. The analyzed risk factors include age, heart disease and neurofibromatosis, number of previous scoliosis surgeries, preoperative Cobb angle, number of fused levels, number of pedicle screws, osteotomy, and operative time. Each risk factor is related to the *p*-value. The dashed vertical line indicates the null effect value (odds ratio = 1.0).

Multivariate logistic regression analysis (Table 5, Figure 2) identified younger age, history of heart disease, greater number of fused levels, and longer operative time as independent risk factors. Each 1-year increase in age reduced the probability of massive blood loss by approximately 17.1% (odds ratio [OR] = 0.829,95% confidence interval [CI], 0.751–0.914, *p* < 0.001). Patients with a history of heart disease had a 4.3 times higher risk of massive blood loss (OR = 4.338, 95% CI: 1.637–11.498, *p* = 0.003). Each additional fused level increased the risk by 11.8% (OR = 1.118, 95% CI: 1.014–1.233, *p* = 0.025). Each additional minute of operative time increased the risk by 0.8% (OR = 1.008, 95% CI: 1.005–1.012, *p* < 0.001).

## 4. Discussion

PSIF is the most common surgical procedure for treating pediatric scoliosis. It improves spinal curvature, alleviates pain, enhances cardiopulmonary function, and improves patient attitudes toward life [18]. Massive intraoperative blood loss remains challenging in PSIF. Multilevel spinal fusion places high demands on surgeons, anesthesiologists, and patients’ physiological reserves. As children have a lower body weight and blood volume than adults, strategies to reduce blood loss, prevent massive hemorrhage, minimize complications, and promote rapid recovery are research priorities. Our study, leveraging a substantial single-center cohort, confirmed that the complexity of the procedure, reflected in a greater number of fused levels and longer operative time, are significant drivers of blood loss. More importantly, we elucidated critical patient-specific factors—younger age and a history of heart disease—that substantially increase a child’s vulnerability, offering new dimensions for preoperative risk stratification.

Because blood volume varies with growth and development in children and adolescents, using absolute blood loss as a metric may introduce bias. This study focused on massive blood loss (EBL/EBV ≥ 30%) [6]. Each 1-year increase in age reduced the probability of massive blood loss by approximately 17.1% (OR = 0.829, *p* < 0.001). This likely reflects the smaller total blood volume and weaker physiological compensatory capacity in younger children. A prior study of 57 congenital scoliosis patients (mean age 8.3 years) similarly found younger age to be a risk factor for increased blood loss, possibly due to richer marrow vasculature [19], while a small series of adolescent idiopathic scoliosis patients (mean age 15.2 years) reported an opposite trend [13]. These contradictory findings highlight the need for large-scale prospective studies across different age groups and etiologies.

Our study identified pre-existing heart disease as a major, independent risk factor, increasing the odds of massive blood loss more than fourfold (OR = 4.338, *p* = 0.003). In a retrospective study of 1601 adolescent patients with scoliosis [20], preexisting heart disease was also identified as an independent risk factor for increased blood loss and transfusion in idiopathic scoliosis surgery, consistent with our findings. However, the underlying reasons were not discussed. In our study, the combined heart diseases mainly included ASD, VSD, PDA and TOF, totaling four types. Notably, a higher proportion of patients in the massive blood loss group had uncorrected cardiac lesions (3/12, 25.0%) compared with the non-massive blood loss group (2/11, 18.2%). These patients often have residual left-to-right or bidirectional shunts, which may prolong the state of excessive pulmonary blood flow and potentially contribute to varying degrees of pulmonary hypertension and elevated right heart pressure [21]. Moreover, the prone position during surgery can impede inferior vena cava return, increasing central venous pressure (CVP), which has been hypothesized to cause dilation and engorgement of paravertebral venous plexuses and, consequently, increased bleeding during dissection and cutting.

These findings can help pediatric spine surgeons and anesthesiologists provide more accurate preoperative counseling for patients and their families regarding the risk of massive blood loss. For such patients, comprehensive preoperative cardiopulmonary evaluation, detailed surgical planning, proactive preparation of blood products, and contingency plans for massive hemorrhage are essential. Intraoperatively, CVP, pulse pressure variation, and stroke volume variation should be monitored for precise volume management. Maintaining a low CVP while ensuring hemodynamic stability may help reduce blood loss in these patients. However, this pathophysiological mechanism remains speculative. Future prospective studies with larger cohorts should include more refined cardiac stratification (e.g., by pulmonary artery pressure, shunt severity, and corrective status) to better elucidate the relationship between specific cardiac conditions and bleeding risk.

Consistent with previous studies [6,20,22,23], we found that a greater number of fused levels was an independent risk factor for massive blood loss. Each additional fused level increased the risk by 11.8%. Two previous studies have suggested that fusion of 12 or more levels predicts massive blood loss [20,22]. A retrospective study of 28,080 pediatric patients with scoliosis indicated that fusion of nine or more levels was associated with a higher likelihood of massive blood loss and increased transfusion requirements. The risk of massive blood loss and transfusion increases with the number of fused levels because more levels require longer incisions, greater exposure, more extensive muscle and soft tissue dissection, and broader bone manipulation.

Similarly, consistent with previous studies [24,25,26], a longer operative time was associated with an increased risk of massive blood loss. Each additional minute of operative time increased the risk by 0.8%. A likely explanation is that prolonged exposure of muscles and bones leads to increased blood loss. Therefore, minimizing operative time without compromising surgical outcomes and safety can reduce the risk of massive blood loss.

A larger Cobb angle has been suggested to be associated with increased blood loss [6,13,27]. However, a study of 114 adolescent patients with idiopathic scoliosis [25] did not find preoperative Cobb angle to be an independent risk factor. Additionally, according to some studies, osteotomy [12] and neurofibromatosis [28,29] are independent risk factors for massive blood loss during pediatric scoliosis surgery. Here, preoperative Cobb angle, osteotomy, and neurofibromatosis were potential risk factors in univariate analysis but not independent risk factors in multivariate analysis. Nevertheless, a larger Cobb angle often indicates greater curve severity and surgical complexity, potentially requiring more fused levels or complex maneuvers. Theoretically, osteotomy and neurofibromatosis (due to underlying vascular abnormalities and coagulopathies) increase blood loss. Thus, vigilance and preparedness for massive blood loss are warranted in these patients. The impacts of these three factors require further confirmation through large-scale prospective studies.

Although several independent risk factors for massive blood loss were identified, TXA use must be carefully considered. Some of our patients received prophylactic TXA; however, the regimen (dose and timing) was not standardized; crucially, TXA was not included as a covariate in the multivariate regression model. This limitation warrants further investigation. Strong evidence indicates that TXA reduces blood loss and transfusion requirements during scoliosis surgery. In a prospective study of 111 adolescent patients with idiopathic scoliosis [30], a TXA loading dose of 50 mg/kg after anesthesia induction followed by a maintenance dose of 10 mg·kg^−1^·h^−1^ reduced intraoperative blood loss by 27%. This effect was most pronounced in surgeries exceeding 4 h and extended into the postoperative period to decrease drainage volume. Johnson et al. [31] demonstrated that high-dose TXA (loading dose 50 mg/kg, maintenance 5 mg·kg^−1^·h^−1^) was more effective than low-dose TXA (loading dose 10 mg/kg, maintenance 1 mg·kg^−1^·h^−1^) in reducing blood loss and transfusion needs in PSIF. Here, the TXA dose used (loading dose 10 mg/kg, maintenance 5 mg·kg^−1^·h^−1^) was relatively low, which may explain why it did not emerge as a protective factor in the statistical analysis.

TXA use may be a significant confounding variable. In clinical practice, surgeons or anesthesiologists may be more inclined to administer TXA to patients perceived to be at higher bleeding risk (e.g., those with more fused levels or in whom longer surgery is anticipated). This nonrandomized decision-making implies that the hemostatic effect of TXA may partially mask the true strength of the association between these inherent risk factors and blood loss. Thus, the ORs for the risk factors identified in this study (e.g., the 4.3-fold risk associated with heart disease) might have been underestimated; the true association could have been stronger than observed. Therefore, our findings regarding TXA should not be interpreted as evidence of its ineffectiveness but as a reflection of the difficulty in accurately assessing its effect in a retrospective design. Future prospective studies should standardize TXA protocols and include TXA as a core variable in the analysis to enable a more unbiased evaluation of other independent risk factors.

Our findings are clinically significant, as these variables help characterize patients at a high risk of massive blood loss during pediatric scoliosis surgery. This knowledge may help clinicians optimize perioperative management to minimize massive blood loss and related complications. However, this study has some limitations.

First, as a single-center retrospective analysis, despite the considerable sample size, the patient population and surgical/anesthetic protocols were relatively homogeneous. Generalizing the conclusions to other institutions requires caution; external validity requires verification through multicenter studies. Second, the retrospective design has inherent limitations. Despite efforts to control for confounders through multivariate regression, unknown or unmeasured confounding factors (e.g., surgeon experience and patient bone density) and the lack of TXA adjustment in multivariate analysis may have affected the accuracy of the results. Third, “heart disease” was broadly defined, and pathophysiological differences among various cardiac conditions were not distinguished; future studies could benefit from a greater number of cases and more detailed classifications. Finally, owing to the retrospective design, some potentially significant intraoperative variables (e.g., specific MAP levels and dynamic changes in CVP) were not systematically collected and analyzed. Prospective multicenter studies with standardized blood management protocols, more precise blood loss measurements, and the inclusion of additional intraoperative physiological parameters are warranted to further validate and refine these results.

## 5. Conclusions

Younger age, history of heart disease, greater number of fused levels, and longer operative time are independently associated with massive intraoperative blood loss in children undergoing PSIF for scoliosis. Early recognition of these factors may help clinicians identify high-risk patients and implement more aggressive blood conservation strategies, including preoperative optimization, proactive preparation of blood products, and meticulous intraoperative hemodynamic management. These measures may contribute to reducing allogeneic transfusion requirements and associated complications, thereby enhancing perioperative safety in this vulnerable population. However, given the retrospective design, these findings reflect associations rather than causal relationships, and prospective multicenter studies are warranted to validate their predictive utility.

## Figures and Tables

**Table 1:** Demographic and clinical characteristics of nonmassive blood loss and massive blood loss groups in patients with scoliosis during surgery (*n* = 460).

Variable	Nonmassive Blood Loss Group (*n* = 334)	Massive Blood Loss Group (*n* = 126)	Z/x^2^	*p*
Age (years)	10.0 (5.0–13.0)	11.0 (8.0–13.0)	2.9	0.003
Sex			1.4	0.242
Male	142 (42.5)	46 (36.5)		
Female	192 (57.5)	80 (63.5)		
Corrected height (cm)	132.5 (108.0–154.3)	140.0 (120.8–157.3)	1.7	0.083
Weight (kg)	27.9 (17.1–45.1)	33.7 (22.3–42.2)	0.9	0.359
BMI (kg/m^2^)	16.2 (14.6–19.0)	16.1 (14.1–18.6)	−1.3	0.194
ASA (*n* (%))			4.4	0.221
I	0 (0)	1 (0.8)		
II	316 (94.6)	115 (91.3)		
III	17 (5.1)	10 (7.9)		
IV	1 (0.3)	0 (0)		
Preoperative Hb (g/L)	133.0 (126.8–141.0)	135.0 ± 11.37	1.3	0.183
Preoperative Hct (%)	39.7 (37.5–42.4)	40.5 ± 3.5	1.8	0.065
Preoperative Cobb angle (°)	35.0 (26.0–45.0)	46.0 (35.0–55.0)	5.1	<0.001
Heart disease (*n*, %)	11 (3.3)	12 (9.5)	7.5	0.006
ASD	4	5		
VSD	5	1		
PDA	1	3		
TOF	0	1		
ASD + VSD	1	2		
Neurofibromatosis (*n*, %)	15 (4.5)	12 (9.5)	4.2	0.041
Number of previous scoliosis surgeries (≥1 time)	72 (21.6)	45 (35.7)	9.7	0.002

Hct, hematocrit; ASD, Atrial septal defect; VSD, Ventricular septal defect; PDA, Patent Ductus Arteriosus; TOF, Tetralogy of Fallot.

**Table 2:** Comparison of Massive Blood Loss Rates Among Surgeons.

Surgeon	Nonmassive Blood Loss Group (*n* = 334)	Massive Blood Loss Group (*n* = 126)	Total	Massive Blood Loss Rate (%)	Z/x^2^	*p*
Surgeon 1	102	41	143	28.7%		
Surgeon 2	89	35	124	28.2%		
Surgeon 3	143	50	193	25.9%		
Total	334	126	460	27.4%	0.357	0.837

**Table 3:** Surgical data of nonmassive blood loss and massive blood loss groups in patients with scoliosis during surgery (*n* = 460).

Variable	Nonmassive Blood Loss Group (*n* = 334)	Massive Blood Loss Group (*n* = 126)	Z/x^2^	*p*
Operative time (min)	192.5 (155.0–310.0)	273.0 (219.0–343.5)	8.3	<0.001
Surgery-related				
Number of fusion segments	6.0 (4.0–12.0)	12.0 (6.0–14.0)	7.3	<0.001
Number of pedicle screws	10.0 (6.0–17.0)	17.0 (10.0–21.3)	6.4	<0.001
Osteotomy (*n*, %)	138 (41.3)	86 (68.3)	26.6	<0.001
Bleeding volume (mL/kg)	11.8 (8.4–16.1)	27.6 (23.4–37.2)	16.4	<0.001
Intraoperative infusion of blood products				
Autologous blood (mL/kg)	6.0 (4.4–8.0)	13.9 (11.9–18.6)	15.7	<0.001
Allogeneic red blood cells (mL/kg)	0 (0–0)	5.6 (0–9.2)	9.5	<0.001
Plasma (mL/kg)	0 (0–0)	0 (0–5.5)	8.6	<0.001
Allogeneic blood transfusion rate (*n*, %)	36 (10.8)	70 (55.6)	103.4	<0.001
Tranexamic acid (*n*, %)	123 (36.8)	37 (29.4)	2.2	0.134
Intraoperative urine volume (mL/kg)	16.1 (11.5–23.8)	19.1 (12.2–27.4)	2.9	0.003

**Table 4:** Postoperative data of nonmassive blood loss and massive blood loss groups in patients with scoliosis during surgery (*n* = 460).

Variable	Nonmassive Blood Loss Group (*n* = 334)	Massive Blood Loss Group (*n* = 126)	Z/x^2^	*p*
Postoperative hospital stay (day)	7.0 (6.0–8.0)	7.0 (6.0–8.3)	2.7	0.008
Postoperative blood transfusion (*n*, %)	4 (1.2)	9 (7.1)	11.8	0.002
Postoperative complications				
Subcutaneous effusion (*n*, %)	16 (4.8)	5 (4.0)	0.1	0.706
Pulmonary complications (*n*, %)	12 (3.6)	2 (1.6)	1.2	0.369
Postoperative destination			5.3	0.075
Orthopedic ward (*n*, %)	334 (100)	124 (98.4)		
ICU (*n*, %)	0 (0)	2 (1.6)		
Postoperative Hb (g/L)	117.5 (110.0–124.0)	114.6 ± 15.7	1.3	0.183
Postoperative Hct (%)	34.9 ± 3.7	33.8 ± 4.5	2.6	0.01
Postoperative Cobb angle (°)	5.0 (3.0–13.0)	15.0 (5.0–25.0)	6.0	<0.001
Major curve correction rate (%)	82.8 (68.7–91.9)	68.5 (55.6–83.3)	−5.2	<0.001

ICU, intensive care unit; Hb, hemoglobin; Hct, hematocrit.

**Table 5:** Univariate and multivariate logistic regression analyses of factors affecting massive blood loss during PSIF in children.

Factors	Univariable Model				Multivariable Model			
	B	OR	95% CI	*p*	B	OR	95% CI	*p*
Age (years)	0.082	1.086	1.043–1.143	0.02	−0.188	0.829	0.751–0.914	<0.001
Heart disease (*n*, %)	1.128	3.091	1.327–7.199	0.009	1.468	4.338	1.637–11.498	0.003
Neurofibromatosis (*n*, %)	0.806	2.239	1.017–4.926	0.045	0.699	2.012	0.836–4.843	0.119
Preoperative Cobb angle (°)	0.027	1.027	1.015–1.040	<0.001	−0.021	1.005	0.990–1.020	0.515
Number of previous scoliosis surgeries (≥1 time)	0.092	1.096	1.003–1.198	0.043	0.005	0.979	0.874–1.096	0.714
Number of fused levels	0.180	1.198	1.138–1.261	<0.001	0.112	1.118	1.014–1.233	0.025
Number of pedicle screws	0.103	1.109	1.074–1.144	<0.001	0.053	1.054	0.982–1.132	0.147
Osteotomy (*n*, %)	1.116	3.054	1.979–4.712	<0.001	0.373	1.452	0.715–2.950	0.302
Operative time	1.010	1.010	1.007–1.012	<0.001	0.008	1.008	1.005–1.012	<0.001

OR, odds ratio; CI, confidence interval.

## Data Availability

The data supporting the findings of this study are available from the corresponding author upon reasonable request due to ethical and privacy restrictions involving pediatric participants.

## Abbreviations

The following abbreviations are used in this manuscript:

PSIF	posterior spinal instrumentation and fusion
EBL	estimated blood loss
EBV	estimated blood volume
OR	odds ratio
CI	confidence interval
ASA	American Society of Anesthesiologists
HR	heart rate
MAP	mean arterial pressure
Hb	hemoglobin
BMI	body mass index
Hct	hematocrit
ASD	Atrial septal defect
VSD	Ventricular septal defect
PDA	Patent Ductus Arteriosus
TOF	Tetralogy of Fallot
TXA	tranexamic acid
ICU	intensive care unit
CVP	central venous pressure
